# Supplementary material for: Fern Spores—“Ready-to-Use” Standards for Plant Genome Size Estimation Using a Flow Cytometric Approach
Source: Plants (Basel). 2022 Dec 27;12(1):140. doi: 10.3390/plants12010140 (PMC9824788; doi:10.3390/plants12010140)
Supplement: Supplementary file 1 [file plants-12-00140-s001.zip › supplementary figures S1-S3.pdf]

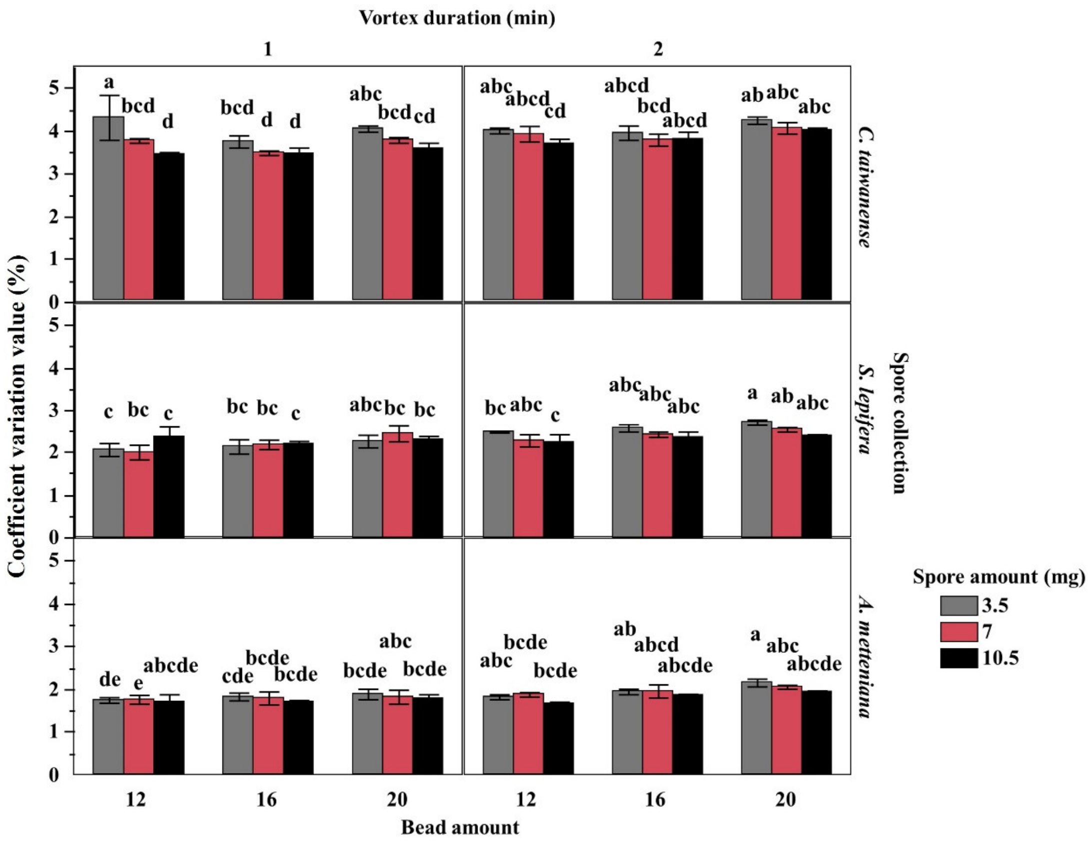

Supplementary Figure S1. Cross-validation values of the three spore collections. Results of Tukey's honest significant difference (HSD) test are labeled with alphabets.

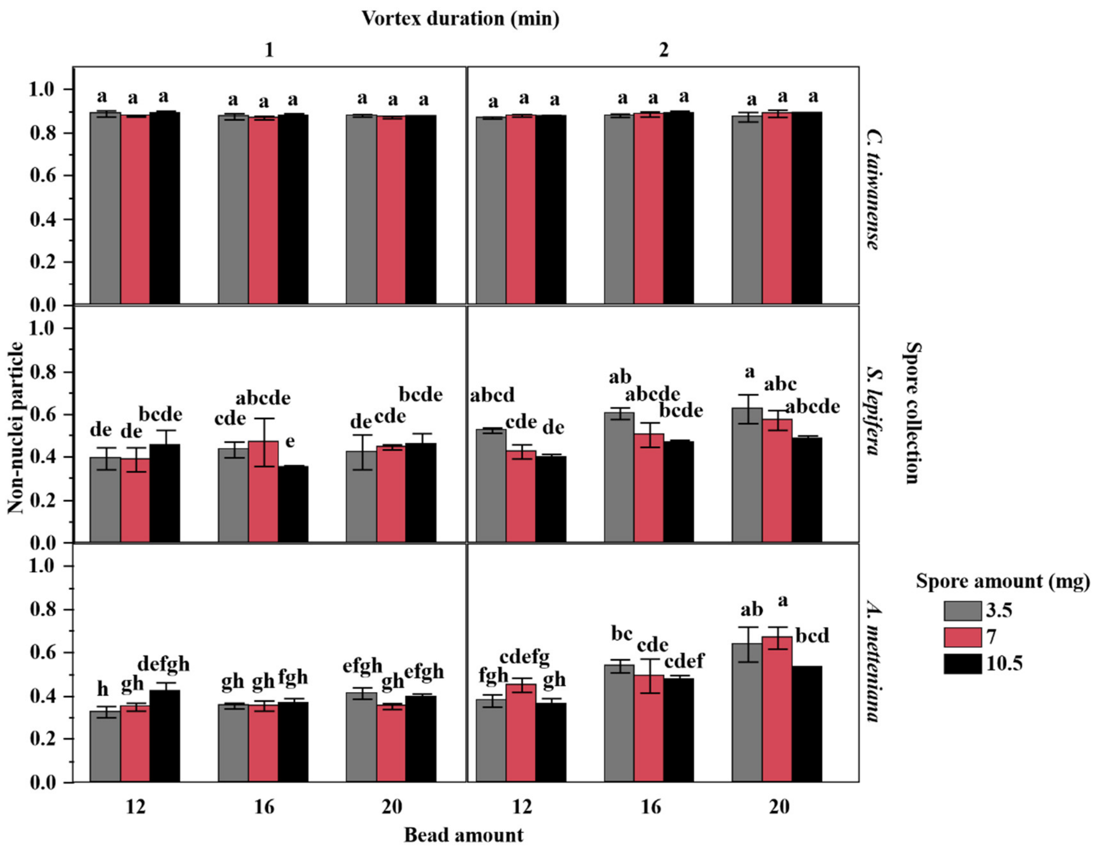

Supplementary Figure S2. Non-nuclear particle values of the three spore collections. Results of Tukey's honest significant difference (HSD) test are labeled with alphabets.

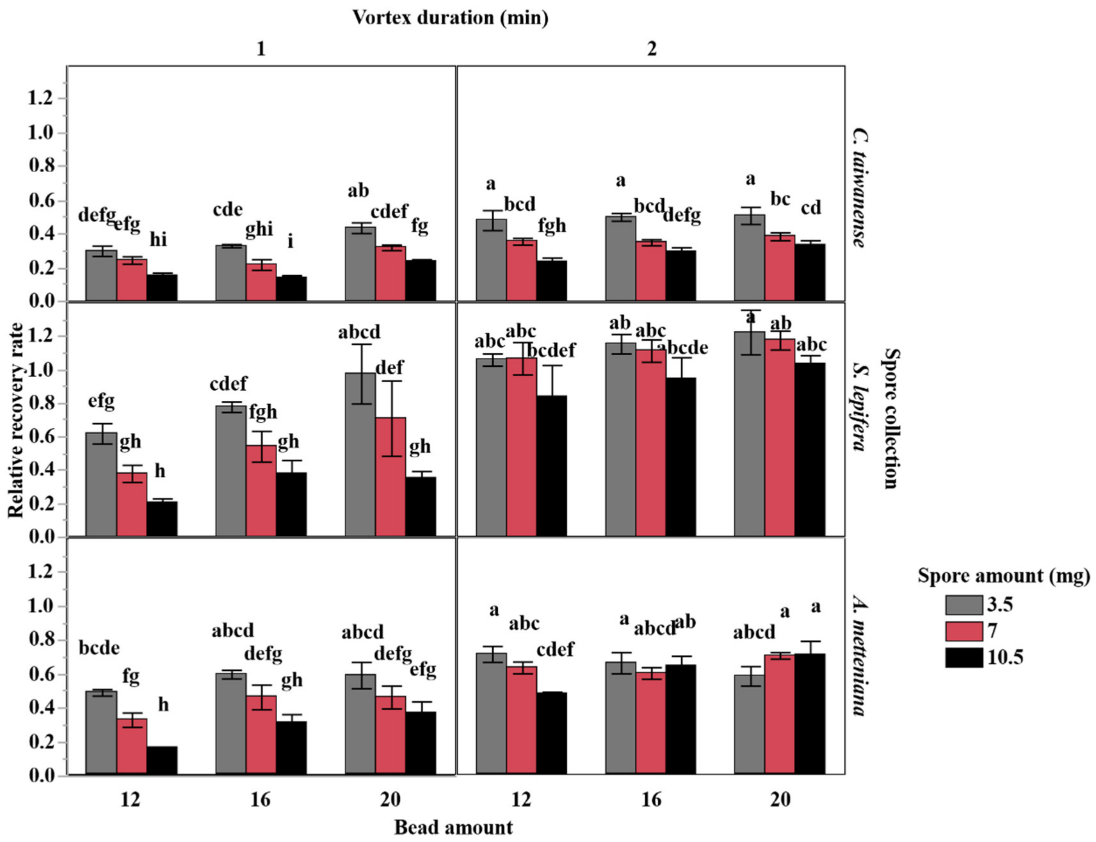

Supplementary Figure S3. Relative recovery rate values of the three spore collections. Results of Tukey's honest significant difference (HSD) test are labeled with alphabets.
