# Supplementary material for: Fern Spores—“Ready-to-Use” Standards for Plant Genome Size Estimation Using a Flow Cytometric Approach
Source: Plants (Basel). 2022 Dec 27;12(1):140. doi: 10.3390/plants12010140 (PMC9824788; doi:10.3390/plants12010140)
Supplement: Supplementary file 1 [file plants-12-00140-s001.zip › supplementary tables S1-S3.pdf]

Supplementary Table S1. Flow cytometric results and collection information of additional *S. lepifera* spore collections. The flow cytometry experiment was conducted during 2019/10/27.

Collection dates	Collection sites	CV (%)	Non-nuclei particle	Relative recovery rate	1C-value
2000/5/28	Yangmingshan, Taipei city, Taiwan	5.54	0.96	0.039	8.34
2006/6/28	Szukanshui, New Taipei city, Taiwan	4.28	0.81	0.189	7.92
2010/8/1	Nanao, Ilan county, Taiwan	3.53	0.78	0.165	7.60
2011/6/8	Suao, Ilan county, Taiwan	4.88	0.93	0.145	8.17
2011/7/14	Kungliao, New Taipei city, Taiwan	4.36	0.91	0.113	7.92
2011/7/26	Lanyu, Taitung County, Taiwan	4.49	0.80	0.104	7.61
2011/8/17	Peitou, Taipei city, Taiwan	4.32	0.78	0.223	7.73
2011/8/27	Machi Industry Road, Keelung city, Taiwan	6.25	0.96	0.113	7.66
2011/9/8	Pingtung, Pingtung country, Taiwan	3.18	0.49	0.363	7.41
2011/9/19	Sanhsia, New Taipei city, Taiwan	4.87	0.77	0.132	7.19
2011/9/30	Pinghsi, New Taipei city,	4.39	0.66	0.171	7.30

	Taiwan				
2011/11/16	Fuchoushan Park, Taipei city, Taiwan	4.96	0.79	0.275	6.90
2012/8/19	Chinshuiying, Taitung County, Taiwan	5.7	0.93	0.22	8.03
2013/6/6	Mt. Hemei, New Taipei city, Taiwan	4.16	0.43	0.342	7.52
2013/8/6	Fushan Botanical Garden, Ilan Country, Taiwan	3.98	0.72	0.132	7.23
2014/4/14	Mt. Hemei, New Taipei city, Taiwan	4.15	0.45	0.351	7.35
2014/8/12	Mt. Hemei, New Taipei city, Taiwan	3.62	0.49	0.271	7.29
2015/4/14	Mt. Hemei, New Taipei city, Taiwan	3.36	0.46	0.369	6.97
2017/6/28	Taipei botanical garden, Taipei city, Taiwan*	3.52	0.52	0.202	7.17
2018/6/10	Taipei botanical garden, Taipei city, Taiwan*	3.5	0.34	0.492	7.16
2019/8/7	Taipei botanical garden, Taipei city, Taiwan	4.76	0.84	0.144	6.95

Supplementary Table S2. Collection information of additional *S. lepifera* spore collections from Herbarium of Taiwan Forestry Research Institute. The flow cytometry experiment was conducted during 2019/10/27.

ID	Collector	Collector no.	Location	Date (Y/M/D)
164871	Bi-Jao Wang	15683	Lanyu, Taiwan	1989/6/4
124095	Unknown	14495	Wulai, Taiwan	1996/8/28
95034	Unknown	Unknown	Wulai, Taiwan	1998/7/28
107382	Tien-Tsai Chen	9763	Lanyu, Taiwan	1999/7/15
119253	Che-Lu Huang	24	Hualin, Taiwan	2000/6/27
133492	Unknown	3376	Huesuen, Taiwan	2001/3/7
374995	Mei-Hsueh Huang, Jen-Jung Teng	mh1406	Sanzhi Visitor Center, Taiwan	2011/9/1

Supplementary Table S3. FCM results with different bead, spore amounts, and bead-vortxing conditions.

Spore	Vortex duration (min)	Spore amount (mg)	Bead amount	Spore:bead	CV	Spore nuclei particle	Total events	Non-nuclei particle	Spore nuclei particle	Standard 2C particle	Relative amount	Spore peak	Standard peak	Nuclei acquaring effeciency
C. taiwanense	1	3.5	12	0.291666667	3.94	1020	8760	0.883561644	1925	1636	1.176650367	195.02	313.36	0.336185819
C. taiwanense	1	3.5	12	0.291666667	4.92	1002	9345	0.892776886	1392	1407	0.989339019	194.79	313.1	0.282668291
C. taiwanense	1	3.5	12	0.291666667	4.08	505	5715	0.911636045	1533	1499	1.022681788	198.5	322.1	0.292194797
C. taiwanense	1	3.5	16	0.21875	3.73	1019	8640	0.882060185	1635	1352	1.209319527	197.81	320.74	0.345519865
C. taiwanense	1	3.5	16	0.21875	3.89	1006	7665	0.868754077	1564	1368	1.143274854	197.76	320.34	0.326649958
C. taiwanense	1	3.5	16	0.21875	3.61	1014	9765	0.896159754	1553	1352	1.148668639	198.11	321.19	0.32819104
C. taiwanense	1	3.5	20	0.175	4.12	1042	8925	0.8832493	2058	1379	1.492385787	197.72	320.1	0.426395939
C. taiwanense	1	3.5	20	0.175	4.03	1015	8715	0.883534137	1985	1348	1.472551929	199.26	323.43	0.420729123
C. taiwanense	1	3.5	20	0.175	3.99	1034	9515	0.89132948	2258	1348	1.675074184	199.05	323.18	0.478592624
C. taiwanense	1	7	12	0.583333333	3.75	1059	9510	0.888643533	2230	1329	1.677953348	199.59	325.23	0.239707621
C. taiwanense	1	7	12	0.583333333	3.73	1200	10065	0.880774963	2628	1367	1.922457937	203.17	331.1	0.274636848
C. taiwanense	1	7	12	0.583333333	3.82	1048	9345	0.887854468	2145	1319	1.626231994	198.34	323.09	0.232318856
C. taiwanense	1	7	16	0.4375	3.49	1018	8625	0.881971014	2310	1356	1.703539823	201.17	328.18	0.243362832
C. taiwanense	1	7	16	0.4375	3.42	1028	7695	0.866406758	2189	1330	1.645864662	201.03	330.8	0.235123523
C. taiwanense	1	7	16	0.4375	3.53	1018	8400	0.878809524	1744	1334	1.307346327	200.45	329.04	0.186763761
C. taiwanense	1	7	20	0.35	3.71	1018	8820	0.884580499	3130	1354	2.311669129	200.16	324.33	0.330238447
C. taiwanense	1	7	20	0.35	3.84	1025	8175	0.874617737	2892	1356	2.132743363	200.79	325.56	0.304677623
C. taiwanense	1	7	20	0.35	3.79	1022	8325	0.877237237	3212	1358	2.365243004	200.27	324.03	0.337891858
C. taiwanense	1	10.5	12	0.875	3.43	1075	9060	0.881346578	2481	1341	1.850111857	201.2	329.95	0.176201129
C. taiwanense	1	10.5	12	0.875	3.42	1016	10050	0.898905473	2087	1334	1.564467766	198.39	323.07	0.14899693
C. taiwanense	1	10.5	12	0.875	3.49	1013	10680	0.905149813	1913	1332	1.436186186	201.36	331.76	0.136779637
C. taiwanense	1	10.5	16	0.65625	3.35	1023	7845	0.86959847	2151	1331	1.616078137	200.55	328.14	0.153912203
C. taiwanense	1	10.5	16	0.65625	3.61	1025	9780	0.895194274	2057	1355	1.518081181	197.46	320.47	0.14457916
C. taiwanense	1	10.5	16	0.65625	3.4	1036	9315	0.888781535	1761	1342	1.312220566	198.53	324.17	0.124973387
C. taiwanense	1	10.5	20	0.525	3.41	1017	8700	0.883103448	3600	1352	2.662721893	205.06	334.22	0.253592561
C. taiwanense	1	10.5	20	0.525	3.63	1024	8970	0.885841695	3307	1353	2.444198078	204.77	332.78	0.232780769
C. taiwanense	1	10.5	20	0.525	3.68	1012	8280	0.877777778	3353	1347	2.489235338	204.79	333.16	0.237070032
C. taiwanense	2	3.5	12	0.291666667	4.08	1035	8100	0.872222222	2390	1377	1.735657226	196.02	317.97	0.495902065
C. taiwanense	2	3.5	12	0.291666667	3.96	1016	8010	0.873158552	2667	1403	1.900926586	193.73	314.64	0.543121882
C. taiwanense	2	3.5	12	0.291666667	3.98	1030	8640	0.880787037	2108	1424	1.480337079	194.32	316.74	0.422953451
C. taiwanense	2	3.5	16	0.21875	4.12	1032	8595	0.879930192	2328	1382	1.684515195	194.52	316.04	0.481290056
C. taiwanense	2	3.5	16	0.21875	3.79	1017	9600	0.8940625	2551	1432	1.781424581	194.47	314.78	0.508978452
C. taiwanense	2	3.5	16	0.21875	3.93	1003	8475	0.881651917	2546	1377	1.848946986	195.94	318.13	0.528270567
C. taiwanense	2	3.5	20	0.175	4.26	1018	9885	0.898098098	2476	1406	1.761024182	196.83	320.84	0.503149766
C. taiwanense	2	3.5	20	0.175	4.33	1030	9990	0.854519774	2810	1408	1.995738636	198.47	322.68	0.570211039
C. taiwanense	2	3.5	20	0.175	4.16	1033	7080	0.884062851	2285	1394	1.639167862	198.42	322.53	0.468333675
C. taiwanense	2	7	12	0.583333333	4.11	1019	8910	0.885634119	3268	1363	2.397652238	199.05	321.88	0.342521748
C. taiwanense	2	7	12	0.583333333	3.75	1061	8940	0.881319911	3717	1396	2.66260745	198.9	322.17	0.380372493
C. taiwanense	2	7	12	0.583333333	3.91	1018	9315	0.890713902	3382	1350	2.505185185	198.94	321.99	0.357883598
C. taiwanense	2	7	16	0.4375	3.71	1018	10290	0.901068999	3319	1367	2.427944404	199.32	322.98	0.346849201
C. taiwanense	2	7	16	0.4375	3.69	1023	9855	0.896194825	3269	1357	2.40899042	201.35	324.96	0.344141489
C. taiwanense	2	7	16	0.4375	3.94	1049	8700	0.879425287	3609	1375	2.624727273	200.83	324.61	0.374961039
C. taiwanense	2	7	20	0.35	3.99	1013	8160	0.910905893	3557	1348	2.638724036	201.13	324.78	0.376960577
C. taiwanense	2	7	20	0.35	4.22	1107	11370	0.89707113	4017	1374	2.923580786	198.49	319.6	0.417654398
C. taiwanense	2	7	20	0.35	3.97	1018	10755	0.878809524	3575	1350	2.648148148	202.24	329.6	0.378306878
C. taiwanense	2	10.5	12	0.875	3.8	1018	8400	0.878809524	3766	1349	2.791697554	203.62	331.95	0.265875957
C. taiwanense	2	10.5	12	0.875	3.58	1024	9165	0.888270595	3352	1382	2.425470333	197.07	320.03	0.230997175
C. taiwanense	2	10.5	12	0.875	3.71	1021	8610	0.881416957	3059	1348	2.269287834	199.6	326.87	0.216122651
C. taiwanense	2	10.5	16	0.65625	3.59	1023	8970	0.885953177	3904	1355	2.881180812	200.16	325.43	0.274398173
C. taiwanense	2	10.5	16	0.65625	3.9	1010	8970	0.894129979	4620	1358	3.402061856	201.81	326.33	0.324005891
C. taiwanense	2	10.5	16	0.65625	3.9	1028	9540	0.906884058	4308	1370	3.144525547	205.26	335.38	0.299478624
C. taiwanense	2	10.5	20	0.525	4.06	1026	11040	0.902425107	4555	1361	3.346803821	204.24	330.49	0.318743221
C. taiwanense	2	10.5	20	0.525	3.94	1027	10515	0.8936853	4701	1355	3.469372694	204.88	330.56	0.330416447
C. taiwanense	2	10.5	20	0.525	4.02	1017	9660	0.894720497	5371	1389	3.866810655	204.69	330.41	0.368267681
S. lepifera	1	3.5	12	0.291666667	2.22	1034	1875	0.448533333	3107	1334	2.329085457	205.37	276.66	0.665452988
S. lepifera	1	3.5	12	0.291666667	2.02	1050	1620	0.351851852	3084	1613	1.911965282	200.32	271.08	0.546275795
S. lepifera	1	3.5	12	0.291666667	1.93	1043	1665	0.373573574	3455	1561	2.213324792	200.73	271.55	0.632378512
S. lepifera	1	3.5	16	0.21875	1.95	1115	1920	0.419270833	3529	1356	2.602507375	201.57	273.28	0.743573536
S. lepifera	1	3.5	16	0.21875	2.15	1124	2130	0.472300469	3670	1354	2.710487445	201.32	274.31	0.774424984
S. lepifera	1	3.5	16	0.21875	2.3	1192	1995	0.402506266	3881	1379	2.814358231	199.64	270.46	0.804102352
S. lepifera	1	3.5	20	0.175	2.4	1180	2220	0.468468468	3973	1457	2.726835964	200.87	274.01	0.77909599
S. lepifera	1	3.5	20	0.175	2.1	1210	1770	0.316384181	5638	1364	4.133431085	197.65	273.3	1.18098031
S. lepifera	1	3.5	20	0.175	2.16	1043	1740	0.400574713	4458	1446	3.082987552	194.24	262.63	0.880853586
S. lepifera	1	7	12	0.583333333	2.13	1186	1815	0.346556474	3444	1349	2.553002224	202.54	287.55	0.364714603
S. lepifera	1	7	12	0.583333333	1.81	1133	1770	0.359887006	4303	1408	3.056107955	203.56	279.19	0.436586851
S. lepifera	1	7	12	0.583333333	2.06	1215	2205	0.448979592	3157	1345	2.347211896	203.17	279.76	0.335315985
S. lepifera	1	7	16	0.4375	2.09	1120	1905	0.412073491	6103	1487	4.104236718	204.58	283.04	0.586319531
S. lepifera	1	7	16	0.4375	2.15	1428	2355	0.393630573	7092	1695	4.184070796	200.05	280.19	0.597724399
S. lepifera	1	7	16	0.4375	2.3	1192	2925	0.592478632	4376	1442	3.034674064	203.57	281.05	0.433524866
S. lepifera	1	7	20	0.35	2.62	1209	2190	0.447945205	5492	1455	3.774570447	202.57	278.87	0.53922435
S. lepifera	1	7	20	0.35	2.5	1034	1830	0.434972678	6462	1385	4.665703971	203.8	281.88	0.666529139
S. lepifera	1	7	20	0.35	2.17	1181	2070	0.429468599	6556	1609	4.074580485	201.48	288.78	0.582082926
S. lepifera	1	10.5	12	0.875	2.64	1048	1995	0.474686717	3244	1351	2.401184308	209.27	299.52	0.22868422
S. lepifera	1	10.5	12	0.875	2.25	1193	2415	0.506004141	3139	1440	2.179861111	199.88	275.14	0.20760582
S. lepifera	1	10.5	12	0.875	2.2	1036	1650	0.372121212	2834	1503	1.885562209	201.71	280.19	0.179577353
S. lepifera	1	10.5	16	0.65625	2.21	1176	1785	0.341176471	7017	1443	4.862785863	204.05	278.54	0.463122463
S. lepifera	1	10.5	16	0.65625	2.1	1134	1725	0.342608696	5628	1451	3.878704342	203.84	280.8	0.369400414
S. lepifera	1	10.5	16	0.65625	2.25	1035	1620	0.361111111	4515	1454	3.10522696	202.91	281.74	0.295735901
S. lepifera	1	10.5	20	0.525	2.33	1110	2040	0.455882353	5617	1484	3.785040431	199	275.42	0.360480041
S. lepifera	1	10.5	20	0.525	2.35	1186	1995	0.405513784	5893	1445	4.078200692	203.91	279.86	0.388400066
S. lepifera	1	10.5	20	0.525	2.2	1119	2265	0.505960265	4746	1494	3.176706827	204	281.98	0.302543507
S. lepifera	2	3.5	12	0.291666667	2.46	1093	2325	0.529892473	4900	1382	3.545586107	204.27	281.24	1.013024602
S. lepifera	2	3.5	12	0.291666667	2.49	1155	2340	0.506410256	5299	1399	3.787705504	204.16	285.53	1.082201573
S. lepifera	2	3.5	12	0.291666667	2.46	1175	2475	0.525252525	5553	1489	3.729348556	203.44	276.49	1.065528159
S. lepifera	2	3.5	16	0.21875	2.56	1176	2745	0.571584699	6373	1527	4.173542895	193.15	262.72	1.192440827
S. lepifera	2	3.5	16	0.21875	2.49	1197	2985	0.598994975	5301	1400	3.786428571	197.61	272.72	1.081836735
S. lepifera	2	3.5	16	0.21875	2.66	1144	3045	0.624302135	6018	1475	4.08	198.21	272.52	1.165714286
S. lepifera	2	3.5	20	0.175	2.78	1146	2640	0.565909091	8397	1756	4.781890661	189.52	280.27	1.366254474
S. lepifera	2	3.5	20	0.175	2.69	1151	3750	0.693066667	7662	1942	3.945417096	197.87	273.58	1.127262027
S. lepifera	2	3.5	20	0.175	2.65	1155	2880	0.598958333	5710	1418	4.026798307	190.18	268.95	1.150513802
S. lepifera	2	7	12	0.583333333	2.12	1715	2790	0.385304659	11005	1409	7.810503903	204.53	280.98	1.115786272
S. lepifera	2	7	12	0.583333333	2.29	1134	2055	0.448175182	9855	1484	6.64083558	205.73	283.75	0.948690797
S. lepifera	2	7	12	0.583333333	2.41	1206	2115	0.429787234	11087	1424	7.785814607	198.31	275.45	1.11225923

S. lepifera	2	7	16	0.4375	2.35	1225	2175	0.436781609	11414	1397	8.170365068	198.99	278.96	1.16719501
S. lepifera	2	7	16	0.4375	2.48	1146	2445	0.531288344	10249	1411	7.263642807	200.29	278.78	1.037663258
S. lepifera	2	7	16	0.4375	2.41	1139	2445	0.534151329	11139	1427	7.805886475	201.73	280.54	1.115126639
S. lepifera	2	7	20	0.35	2.58	1554	3495	0.555364807	15641	1828	8.556345733	199.31	271.44	1.222335105
S. lepifera	2	7	20	0.35	2.48	1364	3570	0.617927171	11549	1483	7.787592717	198.53	276.07	1.112513245
S. lepifera	2	7	20	0.35	2.56	1205	2565	0.530214425	12872	1568	8.209183673	195.55	269.83	1.172740525
S. lepifera	2	10.5	12	0.875	2.28	1921	3090	0.378317152	16107	1462	11.01709986	203.03	280.66	1.049247606
S. lepifera	2	10.5	12	0.875	2.39	1186	2010	0.409950249	11757	1555	7.560771704	206.35	280.78	0.720073496
S. lepifera	2	10.5	12	0.875	2.02	1152	1905	0.395275591	11549	1518	7.608036891	195.97	269.16	0.724574942
S. lepifera	2	10.5	16	0.65625	2.21	1340	2445	0.45194274	16324	1434	11.38354254	193.38	268.82	1.084146908
S. lepifera	2	10.5	16	0.65625	2.47	1207	2310	0.477489177	14244	1587	8.975425331	197.24	270.9	0.854802412
S. lepifera	2	10.5	16	0.65625	2.37	2497	4605	0.457763301	14702	1598	9.200250313	202.05	277.51	0.876214316
S. lepifera	2	10.5	20	0.525	2.39	1364	2715	0.497605893	13915	1363	10.20909758	201.6	279.71	0.972295008
S. lepifera	2	10.5	20	0.525	2.41	1180	2250	0.475555556	17243	1533	11.24787997	197.83	273.2	1.071226664
S. lepifera	2	10.5	20	0.525	2.35	1230	2310	0.467532468	17367	1584	10.96401515	197.9	281.14	1.044191919
A. metteniana	1	3.5	12	0.291666667	1.68	1218	1725	0.293913043	2611	1570	1.663057325	371.96	192.05	0.475159236
A. metteniana	1	3.5	12	0.291666667	1.8	1133	1725	0.343188406	2543	1444	1.761080332	375	194.4	0.503165809
A. metteniana	1	3.5	12	0.291666667	1.75	1142	1710	0.332163743	2472	1518	1.628458498	374.15	194.53	0.465273857
A. metteniana	1	3.5	16	0.21875	1.87	1206	1890	0.361904762	2891	1439	2.009034051	367.8	191.47	0.574009729
A. metteniana	1	3.5	16	0.21875	1.72	1106	1725	0.35884058	3112	1441	2.159611381	367.45	191.3	0.617031823
A. metteniana	1	3.5	16	0.21875	1.87	1121	1695	0.338643068	2905	1449	2.004830918	367.69	191.39	0.572808834
A. metteniana	1	3.5	20	0.175	2.01	1134	1965	0.422900763	2800	1413	1.981599434	363.66	191.68	0.566171267
A. metteniana	1	3.5	20	0.175	1.88	1113	1935	0.424806202	2531	1410	1.795035461	361.79	191.08	0.512867275
A. metteniana	1	3.5	20	0.175	1.76	1172	1890	0.37989418	3322	1428	2.326330532	371.44	194.98	0.664665866
A. metteniana	1	7	12	0.583333333	1.74	1210	1845	0.344173442	3056	1460	2.093150685	378.19	199.29	0.299021526
A. metteniana	1	7	12	0.583333333	1.86	1274	1905	0.331233596	3749	1450	2.585517241	370.93	195.96	0.369359606
A. metteniana	1	7	12	0.583333333	1.66	1149	1815	0.366942149	2949	1456	2.025412088	367.28	192.86	0.289344584
A. metteniana	1	7	16	0.4375	1.82	1173	1815	0.353719008	3817	1450	2.632413793	372.69	196.71	0.376059113
A. metteniana	1	7	16	0.4375	1.92	1228	1965	0.375063613	4908	1348	3.640949555	366.79	192.57	0.520135651
A. metteniana	1	7	16	0.4375	1.63	1131	1680	0.326785714	4724	1444	3.271468144	371.2	196.51	0.467352592
A. metteniana	1	7	20	0.35	1.97	1133	1785	0.365266106	4303	1464	2.93920765	374.81	198.29	0.419886807
A. metteniana	1	7	20	0.35	1.64	1192	1800	0.337777778	5304	1430	3.709090909	365.8	194.17	0.52987013
A. metteniana	1	7	20	0.35	1.83	1175	1800	0.347222222	4053	1426	2.842215989	356.25	193.36	0.406030856
A. metteniana	1	10.5	12	0.875	1.77	1110	1920	0.421875	2381	1412	1.686260623	370.54	197.15	0.16059625
A. metteniana	1	10.5	12	0.875	1.5	1103	1770	0.376836158	2291	1453	1.576737784	369.05	196.23	0.150165503
A. metteniana	1	10.5	12	0.875	1.82	1121	2070	0.458454106	2322	1456	1.59478022	365.91	194.31	0.15188383
A. metteniana	1	10.5	16	0.65625	1.65	1129	1845	0.388075881	5426	1433	3.786461968	373.37	199.36	0.360615426
A. metteniana	1	10.5	16	0.65625	1.72	1148	1785	0.356862745	4132	1447	2.855563234	366.7	195.95	0.271958403
A. metteniana	1	10.5	16	0.65625	1.72	1234	1890	0.347089947	4117	1411	2.917788802	366.71	196.61	0.277884648
A. metteniana	1	10.5	20	0.525	1.87	1260	2115	0.404255319	4572	1506	3.035856574	364.36	194.51	0.289129197
A. metteniana	1	10.5	20	0.525	1.78	1173	1950	0.398461538	5286	1389	3.805615551	364.76	197.87	0.362439576
A. metteniana	1	10.5	20	0.525	1.66	1237	1980	0.375252525	6390	1422	4.493670886	362.07	195.4	0.427968656
A. metteniana	2	3.5	12	0.291666667	1.86	1223	1890	0.352910053	3242	1425	2.275087719	367.12	192.84	0.650025063
A. metteniana	2	3.5	12	0.291666667	1.82	1138	1920	0.407291667	3660	1416	2.584745763	369.55	194.32	0.738498789
A. metteniana	2	3.5	12	0.291666667	1.75	1145	1815	0.369146006	3603	1411	2.55350815	370.38	195.85	0.729573757
A. metteniana	2	3.5	16	0.21875	1.92	1117	2580	0.567054264	3532	1443	2.447678448	364.49	193.18	0.699336699
A. metteniana	2	3.5	16	0.21875	1.89	1199	2430	0.506584362	3700	1552	2.384020619	364.2	192.48	0.681148748
A. metteniana	2	3.5	16	0.21875	2.01	1132	2430	0.534156379	2888	1411	2.046775337	367.71	193.57	0.584792953
A. metteniana	2	3.5	20	0.175	2.06	1184	2595	0.543737958	3070	1708	1.797423888	366.48	194.5	0.513549682
A. metteniana	2	3.5	20	0.175	2.14	1117	3405	0.67195301	3084	1413	2.182590234	369.62	197.15	0.62359721
A. metteniana	2	3.5	20	0.175	2.25	1126	3660	0.692349727	2953	1414	2.088401697	367.53	196.1	0.596686199
A. metteniana	2	7	12	0.583333333	1.84	1239	2100	0.41	6568	1447	4.539046303	366.69	195.57	0.648435186
A. metteniana	2	7	12	0.583333333	1.85	1651	3075	0.463089431	6436	1422	4.526019691	363.15	193.79	0.646574242
A. metteniana	2	7	12	0.583333333	1.94	1157	2175	0.468045977	5848	1419	4.121212121	361.21	193.67	0.588744589
A. metteniana	2	7	16	0.4375	1.77	1139	1905	0.402099738	5687	1414	4.021923621	368.03	198.51	0.574560517
A. metteniana	2	7	16	0.4375	2.05	1184	2625	0.548952381	5688	1412	4.028328612	363.27	195.02	0.575475516
A. metteniana	2	7	16	0.4375	2.03	1165	2415	0.517598344	6259	1409	4.442157559	363.15	194.79	0.634593937
A. metteniana	2	7	20	0.35	2.02	1421	3930	0.638422392	6709	1404	4.778490028	371.88	198.04	0.682641433
A. metteniana	2	7	20	0.35	2.11	1123	4065	0.723739237	7303	1446	5.050484094	369.49	197.34	0.721497728
A. metteniana	2	7	20	0.35	2.02	1185	3240	0.634259259	6828	1414	4.828854314	373.39	198.93	0.689836331
A. metteniana	2	10.5	12	0.875	1.61	1450	2265	0.3598234	7048	1419	4.966878083	362.14	195.01	0.473036008
A. metteniana	2	10.5	12	0.875	1.67	1262	1890	0.332275132	7623	1507	5.058394161	362.56	195.43	0.481751825
A. metteniana	2	10.5	12	0.875	1.7	1173	1905	0.384251969	7242	1496	4.840909091	363.31	196.31	0.461038961
A. metteniana	2	10.5	16	0.65625	1.81	1577	3135	0.496969697	9376	1418	6.61212976	365.85	197.56	0.629726644
A. metteniana	2	10.5	16	0.65625	1.84	1265	2355	0.462845011	10324	1410	7.321985816	365.81	196.59	0.697331982
A. metteniana	2	10.5	16	0.65625	1.89	1217	2265	0.462693157	8787	1445	6.080968858	366.19	197.18	0.579139891
A. metteniana	2	10.5	20	0.525	1.95	1163	2490	0.532931727	11564	1413	8.184005662	370.11	199.8	0.779429111
A. metteniana	2	10.5	20	0.525	1.95	1146	2430	0.528395062	9414	1437	6.551148225	368.19	197.19	0.623918879
A. metteniana	2	10.5	20	0.525	1.89	1195	2535	0.528599606	10644	1426	7.464235624	364.93	195.86	0.710879583
